# Clinical isolates of *Yersinia enterocolitica* Biotype 1A represent two phylogenetic lineages with differing pathogenicity-related properties

**DOI:** 10.1186/1471-2180-12-208

**Published:** 2012-09-17

**Authors:** Leila M Sihvonen, Kaisa Jalkanen, Elisa Huovinen, Susanna Toivonen, Jukka Corander, Markku Kuusi, Mikael Skurnik, Anja Siitonen, Kaisa Haukka

**Affiliations:** 1 Bacteriology Unit, National Institute for Health and Welfare (THL), Helsinki, Finland; 2 Epidemiological Surveillance and Response Unit, National Institute for Health and Welfare (THL), Helsinki, Finland; 3 Department of Bacteriology and Immunology, Haartman Institute, University of Helsinki, Helsinki, Finland; 4 Department of Mathematics and Statistics, University of Helsinki, Helsinki, Finland; 5 Helsinki University Central Hospital Laboratory Diagnostics, Helsinki, Finland; 6 Department of Food and Environmental Sciences, University of Helsinki, Helsinki, Finland

**Keywords:** *Yersinia enterocolitica* biotype 1A, MLST, 16S rRNA gene, *yst* genes, LPS, Phage typing, Human serum complement killing, Bayesian analysis of population structure, Pathogenicity

## Abstract

**Background:**

*Y. enterocolitica* biotype (BT) 1A strains are often isolated from human clinical samples but their contribution to disease has remained a controversial topic. Variation and the population structure among the clinical *Y. enterocolitica* BT 1A isolates have been poorly characterized. We used multi-locus sequence typing (MLST), 16S rRNA gene sequencing, PCR for *ystA* and *ystB*, lipopolysaccharide analysis, phage typing, human serum complement killing assay and analysis of the symptoms of the patients to characterize 298 clinical *Y. enterocolitica* BT 1A isolates in order to evaluate their relatedness and pathogenic potential.

**Results:**

A subset of 71 BT 1A strains, selected based on their varying LPS patterns, were subjected to detailed genetic analyses. The MLST on seven house-keeping genes (*adk, argA, aroA, glnA, gyrB, thrA, trpE*) conducted on 43 of the strains discriminated them into 39 MLST-types. By Bayesian analysis of the population structure (BAPS) the strains clustered conclusively into two distinct lineages, i.e. Genetic groups 1 and 2. The strains of Genetic group 1 were more closely related (97% similarity) to the pathogenic bio/serotype 4/O:3 strains than Genetic group 2 strains (95% similarity). Further comparison of the 16S rRNA genes of the BT 1A strains indicated that altogether 17 of the 71 strains belong to Genetic group 2. On the 16S rRNA analysis, these 17 strains were only 98% similar to the previously identified subspecies of *Y. enterocolitica*. The strains of Genetic group 2 were uniform in their pathogenecity-related properties: they lacked the *ystB* gene, belonged to the same LPS subtype or were of rough type, were all resistant to the five tested yersiniophages, were largely resistant to serum complement and did not ferment fucose. The 54 strains in Genetic group 1 showed much more variation in these properties. The most commonly detected LPS types were similar to the LPS types of reference strains with serotypes O:6,30 and O:6,31 (37%), O:7,8 (19%) and O:5 (15%).

**Conclusions:**

The results of the present study strengthen the assertion that strains classified as *Y. enterocolitica* BT 1A represent more than one subspecies. Especially the BT 1A strains in our Genetic group 2 commonly showed resistance to human serum complement killing, which may indicate pathogenic potential for these strains. However, their virulence mechanisms remain unknown.

## Background

*Yersinia enterocolitica* species has six biotypes (BTs) of which five (1B, 2, 3, 4, 5) contain pathogenic strains. *Y. enterocolitica* ssp. *enterocolitica* consists mainly of the strains of BT 1B, which are considered highly virulent. Low-virulent ssp. *palearctica* encompasses BTs 2–5 and 1A. Since BT 1A strains lack most of the classical virulence markers, this biotype is often considered non-pathogenic. Nevertheless, BT 1A strains are commonly isolated from patients with diarrhoea*.* Reports supporting the pathogenicity of some BT 1A strains comprise clinical data [1-7] and cell experiments [8-10]. A virulence marker commonly found in BT 1A strains is the gene *ystB* encoding heat-stable *Yersinia* enterotoxin B whereas they usually lack the *ystA* gene found from *Y. enterocolitica* 4/O:3 strains. *Yersinia* enterotoxins A and B are homologues to enterotoxins found in enterotoxigenic *E. coli* (ETEC) and *Vibrio cholerae* non-O1 strains [11]. Higher rates of diarrhoea, weight loss, and death have been detected when young rabbits were infected with a *Y. enterocolitica* strain that produces heat-stable enterotoxin compared to the infection with a knock-out mutant [12]. A majority of the *Y. enterocolitica* BT 1A strains possess the *ystB* gene [13] and some excrete heat-stable YstB enterotoxin at 37°C in experimental conditions corresponding to those found in ileum [14,15].

The BT 1A strains are genetically the most heterogeneous of all the *Y. enterocolitica* biotypes [16-19]. They belong to numerous serotypes, with at least 17 having been identified [20]. It has been suggested that BT 1A should be separated into its own subspecies based on genetic differences on a DNA microarray against *Y. enterocolitica* ssp. *enterocolitica* BT 1B strain 8081 [17]. Likewise, a number of other studies utilizing different methods have suggested that *Y. enterocolitica* BT 1A strains could be divided into two main clusters [16,21-25]. However, since the studies have been conducted on different sets of strains, it is impossible to know whether all the methods would divide the strains into two clusters similarly. Recently, two genome sequences of BT 1A strains with no evident structural differences were published [26]. Notable differences between an environmental serotype O:36 and a clinical BT 1A/O:5 strains were the presence of a Rtx toxin-like gene cluster and remnants of a P2-like prophage in the clinical BT 1A/O:5 isolate [26].

BT 1A was the predominant biotype of *Y. enterocolitica* detected among *Yersinia* isolates from human clinical stool samples in Finland in 2006 [27], as also in other European countries [28]. Of the Finnish patients with a BT 1A strain, 90% suffered from diarrhoea and abdominal pain, but only 35% had fever. Furthermore, 3% of the patients had reactive arthritis compared to 0.3% of the controls [7]. We hypothesized that certain BT 1A strains might have a higher pathogenic potential than others. In order to study this, the clinical BT 1A isolates were investigated using multilocus sequence typing (MLST), 16S rRNA sequencing, *yst*-PCR, lipopolysaccharide (LPS) analysis, sensitivity to five yersiniophages and serum killing assay. MLST results were analysed with BAPS (Bayesian Analysis of Population Structure) program, genetic and phenotypic characteristics of the BT 1A strains were compared and statistical analysis was applied to assess their correlation with the symptoms of the patients.

## Results

### Genetic population structure and phylogeny

**Figure 1:**
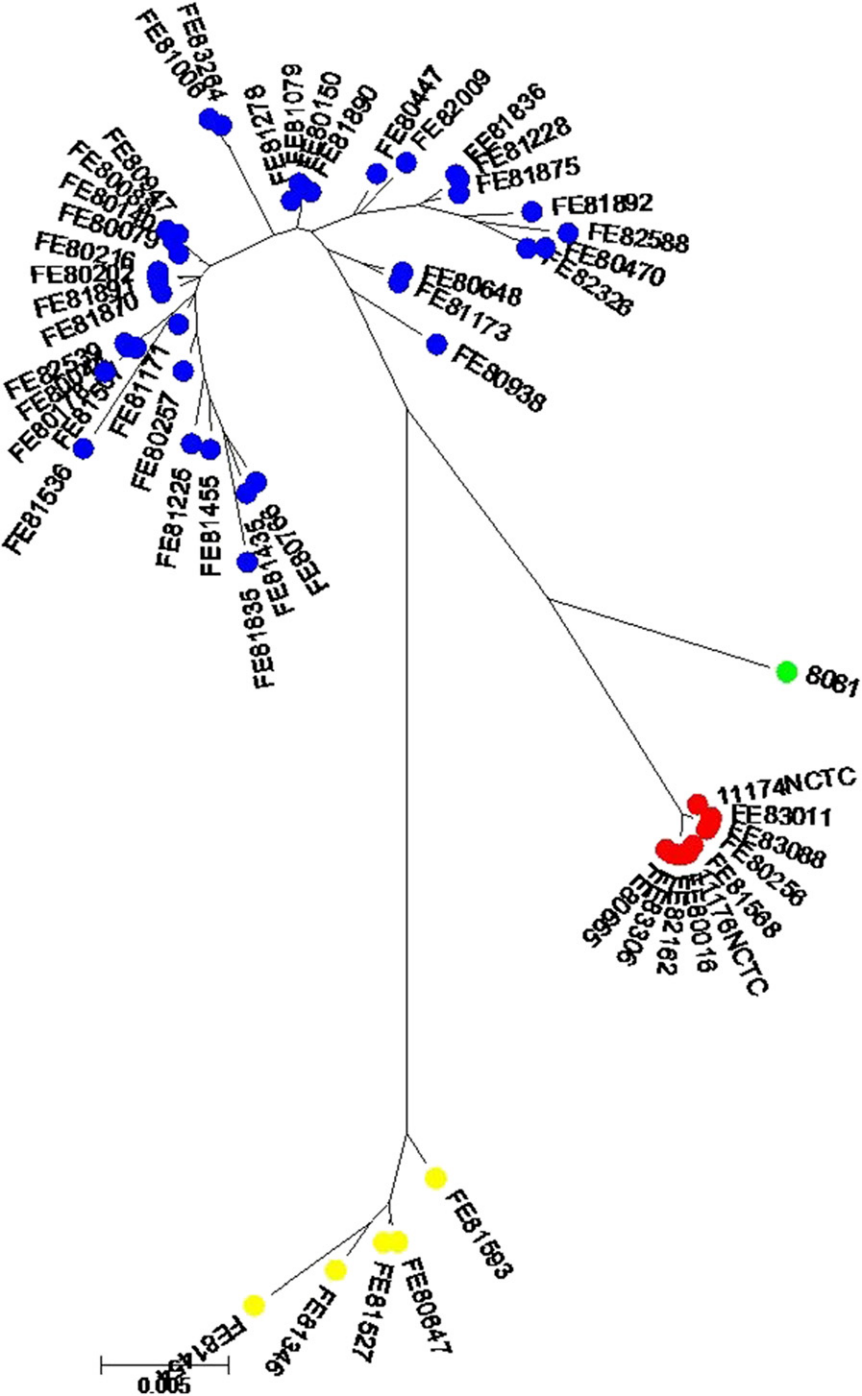
**Maximum likelihood tree based on the MLST of seven house-keeping genes of *Y. enterocolitica* strains.** Color-coding indicates the BAPS groups. The BT 1A strains were divided into two clusters indicated in blue (Genetic group 1) and yellow (Genetic group 2). Strains of BT’s 2–4 are indicated in red and the BT 1B strain in green.

In the MLST analysis, a subset of 43 *Y. enterocolitica* BT 1A strains were discriminated into 39 MLST types and the 10 4/O:3, 3/O:3 or 2/O:9 strains were discriminated into four different MLST types. The genetic diversity indexes for the genes used in MLST were 0.86 (*adk*), 0.93 (*argA*), 0.93 (*aroA*), 0.83 (*glnA*), 0.82 (*gyrB*), 0.94 (*thrA*) and 0.89 (*trpE*). Bayesian analysis of the MLST sequences divided the BT 1A strains into two distinct genetic clusters, which were clearly separated from the tight cluster formed by the strains of BT’s 2–4 and from the BT 1B strain (8018) (Figure 1). One of the BT 1A clusters contained 36 BT 1A and two non-biotypeable strains and was designated as BT 1A Genetic group 1. Another cluster contained five BT 1A strains and was designated as BT 1A Genetic group 2. Ten bio/serotype 3-4/O:3 and 2/O:9 strains clustered closely together, and the single BT 1B strain was located in the vicinity of this cluster. BAPS analysis did not indicate any significant level of mosaicism among the isolates, i.e. no isolates contained variation typical to more than one cluster.

When concatenated MLST sequences (4580 bp) were compared to each other, the BT 1A Genetic group 2 strains were 95–96% similar to BT 1A Genetic group 1, bio/serotype 4/O:3 and 2/O:9, as well as to *Y. enterocolitica* ssp. *enterocolitica* strains of biotype 1B (Table 1). The BT 1A Genetic group 1 strains were 97% similar to bio/serotype 4/O:3 and 2/O:9 and *Y. enterocolitica* ssp. *enterocolitica* strains (Table 1). A neighbour-joining tree depicting the relatedness of the selected *Yersinia* strains and species based on the MLST sequence concatenates is shown in an additional file (Additional file 1).

**Table 1:** Genetic similarity of concatenated seven-gene MLST sequences (4580 bp)

	**BT 1A group1**	**BT 1A group2**	**BT 2–4 O:3/O:9**	**BT 1B 8081**	***Y. kristensenii***	***Y. frederiksenii***	***Y. aldovae***	***Y. rohdei***	***Y. intermedia***	***Y. bercovieri***	***Y. mollaretii***	***Y. ruckeri***
BT 1A Genetic group1	> 99%											
BT 1A Genetic group2	95–96%	> 99%										
BT 2–4 O:3/O:9	97%	95%	> 99%									
BT 1B 8081	97%	95%	98%	100%								
*Y. kristensenii* ATCC 33638	90%	90%	90%	90%	100%							
*Y. frederiksenii* ATCC 33641	87%	87%	87%	87%	86%	100%						
*Y. aldovae* ATCC 35236	87%	87%	87%	87%	87%	85%	100%					
*Y. rohdei* ATCC 43380	86%	86%	86%	86%	85%	86%	84%	100%				
*Y. intermedia* ATCC 29909	85%	85%	85%	85%	86%	86%	86%	84%	100%			
*Y. bercovieri* ATCC 43970	85%	85%	85%	85%	86%	85%	85%	79%	85%	100%		
*Y. mollaretii* ATCC 43969	86%	86%	86%	86%	86%	86%	85%	79%	85%	91%	100%	
*Y. ruckeri* ATCC 29473	79–80%	79%	80%	79%	79%	79%	79%	79%	79%	79%	79%	100%

Comparison of the partial 16S rRNA gene sequences (1310 bp) revealed BT 1A Genetic group 2 strains to have over 99% similarity among themselves, 98–99% similarity to Genetic group 1 strains and 98% similarity to *Y. enterocolitica* BT 2-4/O:3 or O:9 strains (Table 2). Actually, the 16S rRNA gene sequences of BT 1A Genetic group 2 were more similar (99%) to *Y. intermedia, Y. mollaretii, Y. aldovae* and *Y. bercovieri* than to BT 1A Genetic group 1 (Table 2). When the results obtained from representative subsets of 71 strains and analysed using 16S rRNA gene sequencing and MLST were combined, two genetic groups were formed: 17 strains were in Genetic group 2 and 54 in Genetic group 1.

**Table 2:** Genetic similarity of 16S rRNA gene sequences (1310 bp)

	**BT 1A group1**	**BT 1A group2**	**BT2–4 O:3/O:9**	**BT 1B 8081**	***Y. kristensenii***	***Y. frederiksenii***	***Y. aldovae***	***Y. rohdei***	***Y. intermedia***	***Y. bercovieri***	***Y. mollaretii***	***Y. ruckeri***
BT 1A Genetic group1	> 99%											
BT 1A Genetic group2	98–99%	> 99%										
BT 2–4 O:3/O:9	> 99%	98%	> 99%									
BT 1B 8081	99%	98%	99%	100%								
*Y. kristensenii* ATCC 33638	98%	99%	98%	98%	100%							
*Y. frederiksenii* ATCC 33641	98%	98–99%	98%	98%	98.9%	100%						
*Y. aldovae* ATCC 35236	98%	99%	98%	87%	99.2%	98.6%	100%					
*Y. rohdei*ATCC 43380	98–99%	98–99%	98–99%	99.2%	98.8%	99%	98.9%	100%				
*Y. intermedia* ATCC 29909	98%	99%	98%	98%	99%	98.6%	99.4%	98.7%	100%			
*Y. bercovieri* ATCC 43970	98%	99%	98%	98%	98.8%	98.4%	99.2%	98.5%	99.5%	100%		
*Y. mollaretii* ATCC 43969	98%	99%	98%	98%	98.9%	98.6%	99.4%	98.6%	99.4%	99.3%	100%	
*Y. ruckeri*ATCC 29473	97%	98%	97%	97%	98.7%	97.9%	98.1%	97.6%	98%	98.2%	98.2%	100%

**Figure 2:**
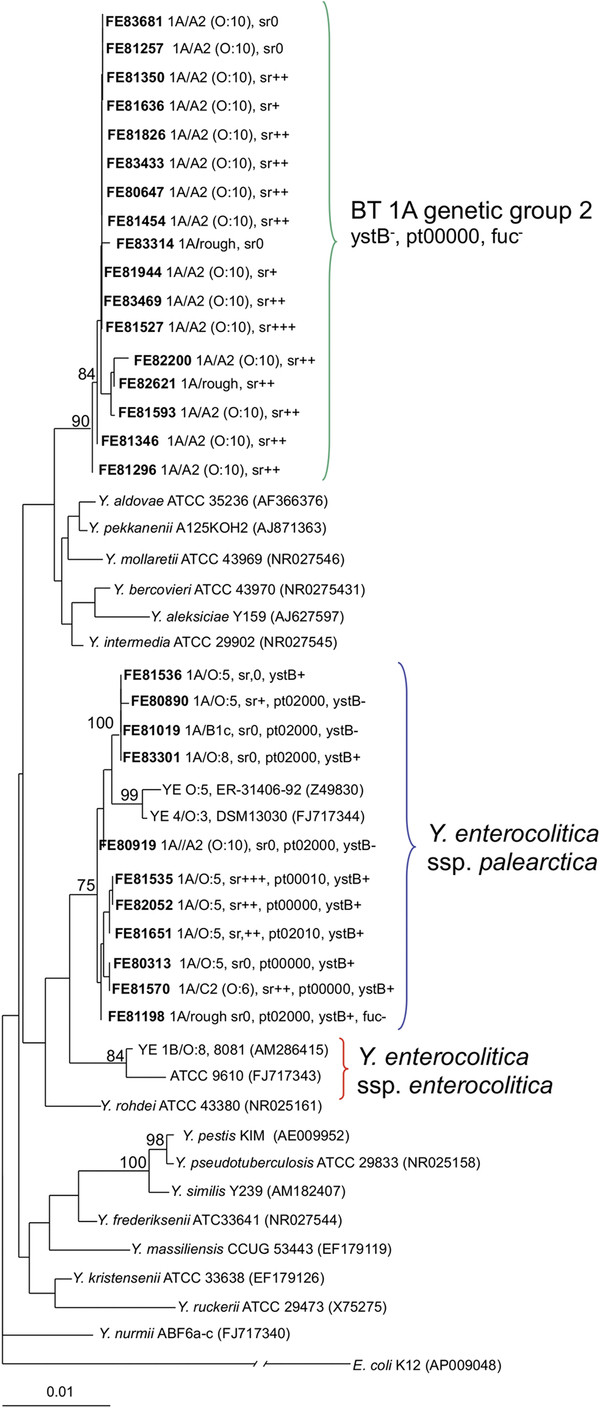
**Neighbor joining tree of 16S rRNA gene sequences (1310 bp) of 47 *Yersinia* strains.** Bootstrap confidence values over 75% (1000 replicates) are given in the branches. sr = serum resistance, pt = phage type, which encodes reaction to 5 phages (φR1–37, PY100, φYeO3–1, φR1-RT, φ80–81). Strains sequenced in the present study are marked bold. Strain ATCC9610 is a type strain of *Y. enterocolitica* ssp. *enterocolitica*.

Of all the BT 1A Genetic group 1 strains included in the MLST analysis, none were *ystA* positive in PCR, but 98% were *ystB* positive. All five of the BT 1A Genetic group 2 strains were both *ystA* and *ystB* negative in PCR. The 4/O:3, 3/O:3 and 2/O:9 strains were all *ystA* positive and *ystB* negative in PCR. When also the BT 1A strains that were not included in the MLST analysis were tested for *ystA* and *ystB*, 12 further strains were found to be negative in *ystB* PCR. They were also subjected to 16S rRNA gene sequencing and were found to be part of BT 1A Genetic group 2 (Figure 2).

### Phenotypic characteristics

Based on the characteristics of the lipopolysaccrarides (LPS) in silver-stained DOC-PAGE gels, the 298 *Y. enterocolitica* BT 1A strains were classified into four main LPS types (A-D), with each containing several subtypes (Table 3). The subtype characteristics are described in detail in an additional file (Additional file 2). Fourteen of the BT 1A Genetic group 2 strains expressed homopolymeric O-PS subtype A2 LPS, similar to LPS of serotype O:10 (Table 3). Three of the BT 1A genetic group 2 strains had rough LPS type. The strains of Genetic group 1 and the non-typeable strains expressed a great variety of LPS types and subtypes. All 77 *Y. enterocolitica* 4/O:3 and 3/O:3 strains included in the LPS analysis expressed homopolymeric subtype A3 O-PS and the five *Y. enterocolitica* 2/O:9 strains subtype A2 O-PS (Table 3). Three of the *ystB* negative strains of BT 1A Genetic group 1 belonged to LPS group A2, two to C1 and one to B1c.

**Table 3:** **LPS types of 298*Y. enterocolitica*BT 1A strains and 84*Y. enterocolitica*strains of other biotypes**

**LPS-type**	**Subtype**	**Description**^**c**^	**Comments**^**d**^	**Known O-serotypes with similar LPS**[56]	**No. of strains (*n*=382)**
*A. Homopolymeric O-PS*					
	A1^a^	Short		O:41(27)43, O:41,43	7
	A2^b^	Medium		O:10	25
	A2	Medium	BT 2 strains	O:9	5
	A3^a^	Long			3
	A3	Long	BT 3–4 strains	O:1, O:2, O:3	77
*B. Heteropolymeric O-PS*					
	B1a ^a^, B1b ^a^	2/M/1	B1b strains carry homopolymer	O:13,18	8
	B1c ^a^, B1d ^a^	2+w/M/1–2	B1d strains carry homopolymer	O:25	9
	B2a ^a^, B2b ^a^	2/L/1	B2b strains carry homopolymer	O:7,8, O:13,7	22
	B2c ^a^, B2d ^a^	2+w/L/1–2	B2d strains carry homopolymer	O:50	55
	B3 ^a^	5–6+w/M/3–6		O:14, O:34, O:4,32	4
	B4 ^a^	>5/M/7–10		O:4, O:8, O:21, O:35,52	1
*C. Single length O-PS*					
	C1 ^a,^	SL 15-mer		O:6, O:6,30, O:6,31	109
	C2 ^a^	SL 30-mer		O:5, O:5,27	45
*D. Rough or semi-rough*					
	D ^a^		May include rough laboratory mutants	O:15, O:28,50, O:35,36	12

^a^ Biotype 1A, Genetic group 1.

^b^ Biotype 1A, Genetic group 2.

^c^ Homopolymeric O-PS length estimated by migration in DOC-PAGE; heteropolymeric O-PS is described by X/Y/Z, where X = number of steps in O-PS ladder (+ w indicates a faint extra step); Y = size of step (M, medium; L, long); Z = average modality of steps; Single length O-PS migrates as one band with estimated number of sugar residues.

^d^ The biotype of the strains is 1A unless otherwise indicated. The presence of homopolymeric O-PS was visible as a smear above the short ladders and not always easy to distinguish in silver-stained DOC-PAGE gels.

Phage sensitivity of the strains was tested using five *Yersinia* specific bacteriophages (Table 4). Most of the 63 bio/serotype 3–4/O:3 strains were sensitive to ϕYeO3-12, PY100 and ϕR1-RT, in addition 7 strains were sensitive to ϕ80-81. The single bio/serotype 2/O:9 strain was infected by ϕR1-RT only. The 273 BT 1A and non-biotypeable strains representing different LPS-types showed variable phage sensitivity patterns further demonstrating the heterogeneity of this group of strains. However, all 17 of the BT 1A Genetic subgroup 2 strains were resistant to all the tested phages.

**Table 4:** **Phage sensitivities of 273*Y. enterocolitica*BT 1A strains and 64*Y. enterocolitica*strains of other biotypes**

**Bio/serotype, LPS-type, Genetic group (number of strains)**	**Number of sensitive strains**				
	**фR1–37**	**PY100**	**фYeO3–12**	**фR1-RT**	**ф80–81**
4/O:3, A3 (n = 63)	0	59	63	61	7
2/O:9, A2 (n = 1)	0	0	0	1	0
1A, A2, Genetic group 1* (n = 16)	2	9	0	1	0
1A, A2, A3 or D1, Genetic group 2* (n = 17)	0	0	0	0	0
1A, A3, Genenetic group 1* (n = 2)	0	1	0	0	0
1A, B1a or B1b (n = 7)	0	2	0	0	0
1A, B1c or B1d (n = 7)	0	6	0	0	0
1A, B2a or B2b (n = 20)	1	11	1	2	1
1A, B2c or B2d (n = 51)	0	51	0	2	20
1A, B3 (n = 3)	0	3	0	0	1
1A, B4 (n = 1)	1	0	0	1	0
1A, C1 (n = 98)	0	38	0	9	0
1A/O:5, C2 (n = 41)	5	30	1	13	0
1A, D1, Genetic group 1* (n = 10)	4	10	1	3	0

*Genetic group 1 or 2 as defined by MLST.

Resistance to human serum complement-mediated killing was most common (99%) in the LPS subtype A3 strains, which included the known pathogenic *Y. enterocolitica* serotype O:3 strains (Table 5). Of the strains in the LPS subtype C2, which included the BT 1A/O:5 isolates, 87% were serum resistant. Serum resistance was also high (67%) among subtype C1 strains, which included BT 1A strains with similar LPS-structure to reference strains of serotypes O:6, O:6,30 and O:6,31. Of the BT 1A LPS subtype A2 (O:10) strains, 72% showed resistance to complement killing. However, 13 of the 14 (93%) BT 1A Genetic group 2 strains among the LPS subtype A2 showed high resistance to complement killing. As a whole, 14 of the 17 (82%) strains of the BT 1A Genetic group 2 were resistant to serum complement killing (Figure 2). Among the LPS B-subtypes, which included a number of the BT1A Genetic group1 isolates, complement resistance was rather low or non-existing (Table 5).

**Table 5:** **Serum resistance distribution among different LPS-types of 298*Y. enterocolitica*BT 1A strains and 83*Y. enterocolitica*strains of other biotypes**

**LPS-type**	**0 (all dead)**	**+ (0.01-5%)**	**++ (5–50%)**	**+++ (> 50%)**	**No. of strains** **(n = 381)**
A1 (O:41(27)43; O:41, 43)^a^	3	2	2	0	7
A2 (O:10) ^d^	6	1	4	1	12
A2 (O:10) Gen. group 2	1	2	9	1	13
A2 (BT 2/O:9)^b^	1	3	1	0	5
A3 (O:1; O:2; O:3)	1	0	0	1	2
A3 (O:1; O:2; O:3) Gen. group 2	1	0	0	0	1
A3 (BT 3–4/O:3)^b^	1	4	25	46	76
B1 (O:13,18; O:25)	10	2	3	2	17
B2 (O:7,8; O:13,7; O:50) ^c^	70	4	2	1	77
B3 (O:14; O:34; O:4,32)	3	1	0	0	4
B4 (O:4; O:8; O:21; O:35,42)	1	0	0	0	1
C1 (O:6; O:6,30; O:6,31)^d^	36	33	35	5	109
C2 (BT 1A/O:5)^b^	6	10	15	14	45
D (rough/semi-rough)	8	1	0	0	9
D (rough/semi-rough) Gen. group 2	1	0	2	0	3

The strains belong to biotype 1A and Genetic group 1 unless otherwise indicated.

^a^ The known serotypes with similar LPS structure shown in parenthesis.

^b^ Serotype confirmed with agglutination test.

^c^ Serotype confirmed with O:8 agglutination test for 56 strains.

^d^ This group contains one non-biotypeable *Y. enterocolitica* strain.

### Statistical analysis of patient symptoms

The symptoms (diarrhoea, vomiting, fever, abdominal pain and blood in stools) of patients with BT 1A did not differ significantly when the statistical analyses were based on the genetic grouping or serum resistance of the BT 1A isolates. The patients with isolates belonging to different LPS-groups were symptomatic, but due to the small amount of patients in analyses, no significant statistical inference could be made.

## Discussion

The strains previously identified by phenotypic tests to belong to *Y. enterocolitica* BT 1A [27] formed two phylogenetic clusters based on MLST analysis, Genetic groups 1 and 2. BT 1A Genetic group 1 comprised of isolates with related 16S rRNA gene sequences but with great variation in their pathogenicity-associated properties. On the contrary, BT 1A Genetic group 2 was found to be rather uniform and phylogenetically distinct from the other *Y. enterocolitica* BT 1A strains. The genetic similarity of this group to Genetic group 1 was 95–96% based on the MLST sequences and 98–99% based on the 16S rRNA gene sequences. All the 17 strains determined to belong to *Y. enterocolitica* BT 1A Genetic group 2 were *ystB* negative in PCR and were resistant to the five tested yersiniophages. Additionally, none of them fermented fucose, as determined in our previous study [27]. Likewise, pathogenic pYV + yersinia strains do not ferment fucose, whilst 91% of the BT 1A strains other than those of Genetic group 2 do. Of the Genetic group 2 strains 82% were resistant to serum complement killing and 76% belonged to LPS type A2.

Remarkably, the 16S rRNA sequences of BT 1A Genetic group 2 were more similar to *Y. intermedia, Y. mollaretii, Y. aldovae* and *Y. bercovieri* than to *Y. enterocolitica* 16S rRNA sequences. However, a previous study indicated that the use of MLST of house-keeping genes determined genetic relatedness among *Yersiniae* better than 16S rRNA [29]. Studies using both DNA hybridization and 16S rRNA gene sequence data have illustrated that if two strains show less than 97% 16S rRNA gene sequence similarity, they are separate species [30]. Nevertheless, even 99% similarity of 16S rRNA genes does not guarantee that bacterial strains represent the same species. Howard and colleagues [17] have already suggested that BT 1A strains should be designated as a third subspecies of *Y. enterocolitica* based on the comparison of whole genomes using DNA microarray. It is likely that the genetic difference between the two phylogenetic groups of *Y. enterocolitica* BT 1A discovered in the present study may also be high enough to justify designation of different subspecies or even species. Although further analyses would be needed for species designation, our data add insight into the phylogeny of the genus *Yersinia*, which is continuously evolving: three novel *Yersinia* species, *Y. entomophaga, Y. pekkanenii* and *Y. nurmii* were described as recently as 2010 [31-33].

This is the first time that two phylogenetic clusters of *Y. enterocolitica* BT 1A strains are reported based on the sequence analysis of house-keeping genes, but similar results indicating the existence of two main clusters of BT 1A strains have been obtained with other molecular methods, such as ribotyping and REP-ERIC [21], *gyrB*-RFLP [22], AFPL [16], MLEE [23,24] and, most recently, MALDI-TOF mass spectrometry to identify the protein mass patterns [25]. Gulati and Virdi [22] found in their study that sequences of *gyrB* genes of representative two clonal groups of BT 1A strains were only 97% similar. When we compared these *gyrB* sequences to our data, sequences DQ140396 and DQ140397 [22] were clustered with BT 1A Genetic groups 1 and 2 of our study, respectively. This is further justification for the separation of BT 1A strains into two phylogenetic lineages. As in our study, the presence of *ystB* gene correlated with the clonal groups, except in one strain [34]. The lack of the *ystB* gene in PCR test does not always correlate with the phylogenetic lineages, since our study also found six strains without the *ystB* gene in BT 1A Genetic group 1. However, only the use of hybridization analysis or sequencing would confirm the PCR results. In a recent study of the whole genome sequences no evident structural difference was found with *ystB*-positive BT 1A/O:5 and BT 1A/O:36 strains [26]. Therefore, it is likely that the two whole genome sequences represent one of the genetic groups of BT 1A of the present study. Blast searches showed that the sequences we obtained for Genetic group 1 were nearly identical with the ones from the above mentioned whole genome sequences, while for Genetic group 2 no matching sequences were detected.

We used DOC-PAGE based classification of LPS to subtype our *Y. enterocolitica* strains. This method offered a practical substitute for O-serotyping, since there are no commercial O-specific antisera available for numerous *Y. enterocolitica* serotypes. The results were consistent with earlier O-serotyping of the BT 1A strains using available commercial antisera [27] which demonstrated that 42 subtype C2 strains were of serotype O:5 and that 56 subtype B2 strains agglutinated with anti-O:8 antiserum indicating that they probably were of the common serotype O:7,8. However, the strains with O:8 antigen, were found in LPS subgroups B2c and B2d which indicates that the classification of subgroups of B2 was tentative and differences could also be inherent to the silver staining procedure. The clinical BT 1A strains showed a wide diversity in their LPS types and this is most likely also reflected in their O-serotypes. The majority of the strains, 37%, had LPS subtype C1 that is similar to that of serotypes O:6,30 and O:6,31, and 15% of the strains had subtype C2, i.e., that of serotype O:5. Globally, the serotypes O:6 and O:5 have been the dominant serotypes of BT 1A associated with diarrhoea [20]. In the present study the strains of LPS subtype C1 and C2 as well as the strains of BT 1A Genetic group 2, demonstrated significant resistance to complement killing, which suggests that the strains of these subgroups may have more pathogenic potential than the other studied strains. Bacterial pathogens have several strategies to resist host defence mechanisms, including resistance to the bactericidal activity of the human serum complement [35]. Pathogenic *Y. enterocolitica* 4/O:3 strains are able to resist serum killing by YadA- and Ail-mediated binding of the serum complement regulatory proteins factor H and C4 binding protein [36-38]. The BT 1A strains of the present study did not possess the *yadA* gene and only one strain had the *ail* gene [39]. Thus the resistance to complement killing of these BT 1A strains must have another, unresolved mechanism.

Although the potential pathogenicity of BT 1A strains remains controversial, there are a few studies that show an association to disease. For instance, BT 1A/O:6,30 was associated with spondyloarthropaties of patients in England and South-Wales [5]. Also, in a study of antibody production, it was found that a patient with symptoms of diarrhoea and reactive arthritis had IgG, IgA and IgM antibodies against the BT 1A/O:6 strain isolated from her fecal sample [6]. We found symptomatic patients with isolates of both BT 1A genetic groups, but did not find statistical differences between the genetic groups and the clinical picture of the symptoms of these patients. It may be that the patients’ genetic or other factors such as gut environment are relevant in the disease caused by BT 1A strains.

## Conclusions

The results of our study present strong evidence that strains classified as *Y. enterocolitica* BT 1A represent more than one subspecies. BT 1A Genetic group 1 consisted of strains with a variety of pathogenicity-related properties, whereas all 17 strains of BT 1A Genetic group 2 lacked the *ystB* gene, belonged either to the same LPS subtype or were rough, were all resistant to the five tested yersiniophages and were largely resistant to serum complement killing. Furthermore, none of them fermented fucose. Although several studies have been conducted to reveal the significance of the BT 1A strains in causing disease, indisputable results have not been obtained. This study shows, however, that BT 1A is a very heterogenous group of strains, some of which might be potential pathogens. Therefore, better understanding of the genetic and phenotypic variability and clustering of these strains, as achieved in our study, would be crucial in determining the pathogenic role of the strains belonging to the defined clusters.

## Methods

### Bacterial strains

Altogether 298 BT 1A, 75 bioserotype 4/O:3, two 3/O:3, five 2/O:9 and two non-biotypable *Y. enterocolitica* strains isolated in 2006 from human samples [27] were utilized in the study. Only one strain per person was included in the study.

### MLST sequencing

MLST analysis was done on 53 *Y. enterocolitica* strains (43 BT 1A and 10 BT’s 2–4 strains) that represented various LPS patterns. Additionally, two reference strains, NCTC11174 (O:9) and NCTC11176 (O:3), were included in the analysis. Genomic DNA was extracted using Jetflex Genomic DNA purification kit (Genomed, Löhne, Germany). Fragments of seven house-keeping genes (*adk, argA, aroA, glnA, gyrB, thrA, trpE*) were amplified by PCR. For the *adk, argA, aroA, glnA, thrA* and *trpE* genes, the primers available in the MLST database for *Y. pseudotuberculosis* at the ERI, University College Cork, were used ( http://mlst.ucc.ie/mlst/dbs/Ypseudotuberculosis/documents/primersPseudotuberculosis_html). The *gyrB* gene amplification was done with the primers described earlier [29]. The 25 μl amplification reactions consisted of 0.25 μM of primers, 0.2 mM dNTP, 2.5 U AmpliTaq Gold (Applied Biosystems, Foster City, USA) and 10 × buffer supplied with the enzyme. The thermal cycle consisted of 10 min denaturation at 94°C, followed by 35 cycles of denaturation for 30 s at 94°C, annealing for 30 s at 51°C, and elongation for 30 s at 72°C and finally for 3 min at 72°C. The PCR fragments were sequenced in both directions with an ABI 3730xl DNA Analyzer (Applied Biosystems). The Diversity indexes for each MLST gene were calculated by eBURST v3 [40,41]. The MLST sequences of 53 *Y. enterocolitica* strains obtained in the study were deposited to EMBL/GenBank database under the accession numbers HE803367- HE803737.

### Analysis of the MLST data

Population genetic analyses were performed using BAPS (Bayesian Analysis of Population Structure) software [42-44] with the second-order Markov model and the standard MLST data input option as in, e.g., [45,46]. The optimal number of clusters was calculated using 10 runs of the estimation algorithm with the prior upper bound of the number of clusters varying in the range (5,15) over the 10 replicates. All estimation runs resulted in an identical partition of the sequence type data with 4 clusters (estimated P = 1.000). Admixture analysis was done using 100 Monte Carlo replicates for allele frequencies and by generating 100 reference genotypes to calculate p-values. For reference cases we used 10 iterations in estimation according to the guidelines of [44,47].

Mosaicism is defined as sequence types composed of sequence characteristic of more than one BAPS group. Significance of admixture or mosaicism was determined for each sequence type using the threshold p < 0.05.

Maximum likelihood tree was constructed by using the concatenated sequences under the general time-reversible model as implemented in the MEGA5 software [48].

### 16S RNA gene sequencing and tree construction

16S rRNA gene sequencing was obtained for 36 *Y. enterocolitica* BT 1A strains with the primers FD1mod [49], pHr, pDf, and pEr [50] in conditions described earlier [22]. The sequences were used to construct a Neighbour-joining tree using Phylip [51]. The 16S rRNA gene sequences of 28 *Y. enterocolitica* BT 1A strains obtained during this study were deposited to the EMBL/GenBank database under the accession numbers HE803738 - HE803765. Eight of the BT 1A strains were sequenced during our previous studies and have accession numbers FM958217 - FM958223 and FN812721 [27].

### *ystA* and *ystB* PCR

For the *ystA* gene specific PCR the forward primer 3-ATC GAC ACC AAT AAC CGC TGAG −5 and reverse primer 3- CCA ATC ACT ACT GAC TTC GGCT −5 were used for 38 *Y. enterocolitica* BT 1A strains. For the *ystB* gene specific PCR the forward primer 3-GTA CAT TAG GCC AAG AGA CG −5 [52] and the reverse primer 3-GCA ACA TAC CTC ACA ACA CC −5 [53] were used for 298 *Y. enterocolitica* BT 1A strains. Chromosomal DNA was used as a template; the conditions for PCR amplification were as described earlier [52].

### DOC-PAGE analysis of LPS

LPS samples of 298 *Y. enterocolitica* BT 1A strains were prepared by the small scale proteinase K method as described earlier [54]. Briefly, the bacteria were grown for 14–16 h with shaking in 2 ml of LB at 22°C (RT); the OD_600_ was determined, the bacteria were then pelleted by centrifugation, and the pellet was re-suspended in DOC lysis buffer (2% DOC, 4% 2-mercaptoethanol, 10% glycerol and 0.002% bromophenol blue in 1 M Tris–HCl buffer, pH 6.8) in a volume adjusted according to the density of the culture (i.e., 100 μl / OD_600_ =1). The suspension was heated to 100°C for 10 min and then 2–4 μl of proteinase K (20 mg/ml) was added and the suspension was incubated overnight at 60°C. An aliquot of 10 μl was loaded on the gel and analysed in 12% DOC-PAGE and the LPS bands were visualized by silver staining as described earlier [55]. The DOC-PAGE-based LPS classification of *Y. enterocolitica* and *Y. enterocolitica* –like bacteria has been described elsewhere [56]. Briefly, based on the O-polysaccharide (O-PS) the strains are classified into four main LPS types: (i) type A, LPS with homopolymeric O-PS, (ii) type B, LPS with ladder-forming heteropolymeric O-PS, (iii) type C, LPS with single-length O-PS, and (iv) type D, rough or semi-rough LPS without O-PS or with a lipid A core substituted with a single O-repeat unit, respectively.

### Phage sensitivity assay

The following bacteriophages were used in the typing scheme: фR1–37 [57,58] that infects *Yersinia* expressing the outer core hexasaccharide in LPS; PY100 that infects a broad range of *Yersinia* strains [59]; фYeO3–12 that uses the *Y. enterocolitica* serotype O:3 O-PS as receptor [60,61]; ϕR1-RT that is a bacteriophage originating from the sewage of Turku, Finland and infects *Y. enterocolitica* serotype O:3 grown at RT (Skurnik, unpublished); and ф80–18 that is a serotype O:8 O-PS specific phage [62]. For altogether 273 *Y. enterocolitica* BT 1A strains, a 40 μl aliquot from a bacterial culture grown for 14–16 h at RT or 37°C with shaking in LB was mixed with 3.5 ml of molten 0.4% soft agar adjusted to 50°C, mixed briefly and poured on an LA plate. After the soft agar had solidified, 10 μl drops of different phage suspensions (~10^5^ plaque forming units ml^-1^) were pipetted onto the surface and the plates were incubated at RT or 37°C 14–16 h. Phage sensitivity was scored as a clear lysis zone in the soft agar.

### Complement killing assay

Blood was obtained from healthy human donors who were devoid of anti-*Yersinia* antibodies. Sera were pooled and stored in aliquots at −70°C. The killing assay for 298 *Y. enterocolitica* BT 1A strains was performed essentially as described previously [63]. Briefly, for bactericidal assay, bacteria were grown to stationary phase overnight in 5 ml of MedECa (MedE: 0.1 g l^-1^ MgSO_4_ × 7H_2_O, 2 g l^-1^ citric acid, 10 g l^-1^ K_2_HPO_4_, 3.5 g l^-1^ NaNH_4_HPO_4_ × 4H_2_O and 1 mg l^-1^ vitamin B_1_, supplemented with 0.2% glucose, 0.2% casamino acids and 2.5 mM CaCl_2_) at 37°C without shaking. The cultures were diluted 1:1000–5000 into PBS to obtain a suspension of ca. 10^5^ cfu/ml and 10 μl of the suspension was mixed with 20 μl of normal human serum (NHS) or heat-inactivated serum (HIS, 30 min at 56°C). After 60 min incubation at 37°C, the complement reaction was stopped by transferring the tubes on ice and the addition of 70 μl of ice-cold BHI. Aliquots of 20 μl were cultured on LA-plates and the surviving bacteria were counted after 48 hr incubation at RT. The serum bactericidal effect was calculated as the survival percentage taking the bacterial counts obtained with bacteria incubated in HIS as 100%. The survival was scored as follows: >50% survival, +++; 5–50% survival, ++; and 0.01–5% survival, +; and no colonies, 0.

### Statistical analysis of the symptoms of the patients

We compared the symptoms of diarrhoea, vomiting, fever, abdominal pain and blood in stools among 98 patients with a *Y. enterocolitica* BT 1A isolate, who had answered a questionnaire about the symptoms [7] and had less than six weeks from the onset of symptoms to the sample-taking. Comparisons (Fischer’s exact test) were done among these patients separately for BT 1A genetic groups 1 and 2 (n = 94 and n = 4); for LPS groups: A1-A3 (n = 5), B1-B4 (n = 41), C1 (n = 37), C2 (n = 10), D1 (n = 5); and for serum resistance groups (n = 46 and n = 52). Analyses were done with STATA 9.0.

### Ethical considerations

Informed consent was obtained from the patients who participated in the questionnaire study. The study was approved by the Ethics Committee of National Institute for Health and Welfare (THL). The voluntary healthy blood donors whose sera were used in serum-killing assay gave their verbal consent. They were informed of the details of the study and their blood samples were pooled and used for the study without an individual being identified.

## Competing interests

The authors’ declare that they have no competing interests.

## Authors’ contributions

LMS conducted the MLST work, combined all the results together and drafted the manuscript. KJ contributed to the genomic analyses. ST and MS conducted and analyzed the LPS, serum resistance and phage typing assays. EH and MK analysed the clinical data and JC did the BAPS and phylogenetic analysis of the MLST data. AS and KH participated in planning of the work, analyzing the results and writing the article. All authors read and approved the final manuscript.

## Supplementary Material

Additional file 1 **Neighbour-joining tree based on seven concatenated MLST genes (4580 bp).** Neighbour-joining bootstrap confidence values over 75% (1000 replicates) are given in the branches. BT 1A strains were *ystB* positive in PCR and had positive reaction in fucose fermentation unless otherwise indicated. sr=serum resistance; pt= phage type, which encodes reaction to 5 phages (φR1–37, PY100, φYeO3–1, φR1-RT, φ80–81). Strains sequenced in the present study are marked bold. In addition, the following GenBank sequences were used: *Y. enterocolitica* 8081 (AM286415), *Y. aldovae* ATCC 35236 (ACCB00000000), *Y. kristensenii* ATCC 33638: (ACCA00000000), *Y. intermedia* ATCC 29909 (AALF00000000), *Y. frederiksenii* ATCC 33641 (AALE00000000), *Y. mollaretii* ATCC 43969 (AALD00000000), *Y. bercovieri* ATCC 43970 (AALC00000000), *Y. rohdei* ATCC 43380 (ACCD00000000) and *Y. ruckeri* ATCC 29473 (ACCC00000000). Click here for file

Additional 2 **Analysis of *Y. enterocolitica* LPS by DOC-PAGE and silver staining.** The picture is compiled of gel images with different LPS types as indicated above the lanes by the LPS type codes that are explained in the text box. Please note that LPS types A2, B1c, B1d, B2a, B2c and B4 are not shown. The gel regions where O-PS and lipid A (LA) plus core migrate are indicated by arrows. Click here for file
