# Supplementary material for: Clinical isolates of Yersinia enterocolitica Biotype 1A represent two phylogenetic lineages with differing pathogenicity-related properties
Source: BMC Microbiol. 2012 Sep 17;12:208. doi: 10.1186/1471-2180-12-208 (PMC3512526; doi:10.1186/1471-2180-12-208)
Supplement: Additional file 1 — Neighbour-joining tree based on seven concatenated MLST genes (4580 bp). Neighbour-joining bootstrap confidence values over 75% (1000 replicates) are given in the branches. BT 1A strains were ystB positive in PCR and had positive reaction in fucose fermentation unless otherwise indicated. sr=serum resistance; pt= phage type, which encodes reaction to 5 phages (φR1–37, PY100, φYeO3–1, φR1-RT, φ80–81). Strains sequenced in the present study are marked bold. In addition, the following GenBank sequences were used: Y. enterocolitica 8081 (AM286415), Y. aldovae ATCC 35236 (ACCB00000000), Y. kristensenii ATCC 33638: (ACCA00000000), Y. intermedia ATCC 29909 (AALF00000000), Y. frederiksenii ATCC 33641 (AALE00000000), Y. mollaretii ATCC 43969 (AALD00000000), Y. bercovieri ATCC 43970 (AALC00000000), Y. rohdei ATCC 43380 (ACCD00000000) and Y. ruckeri ATCC 29473 (ACCC00000000). [file 1471-2180-12-208-S1.docx]

**Additional file 1.**


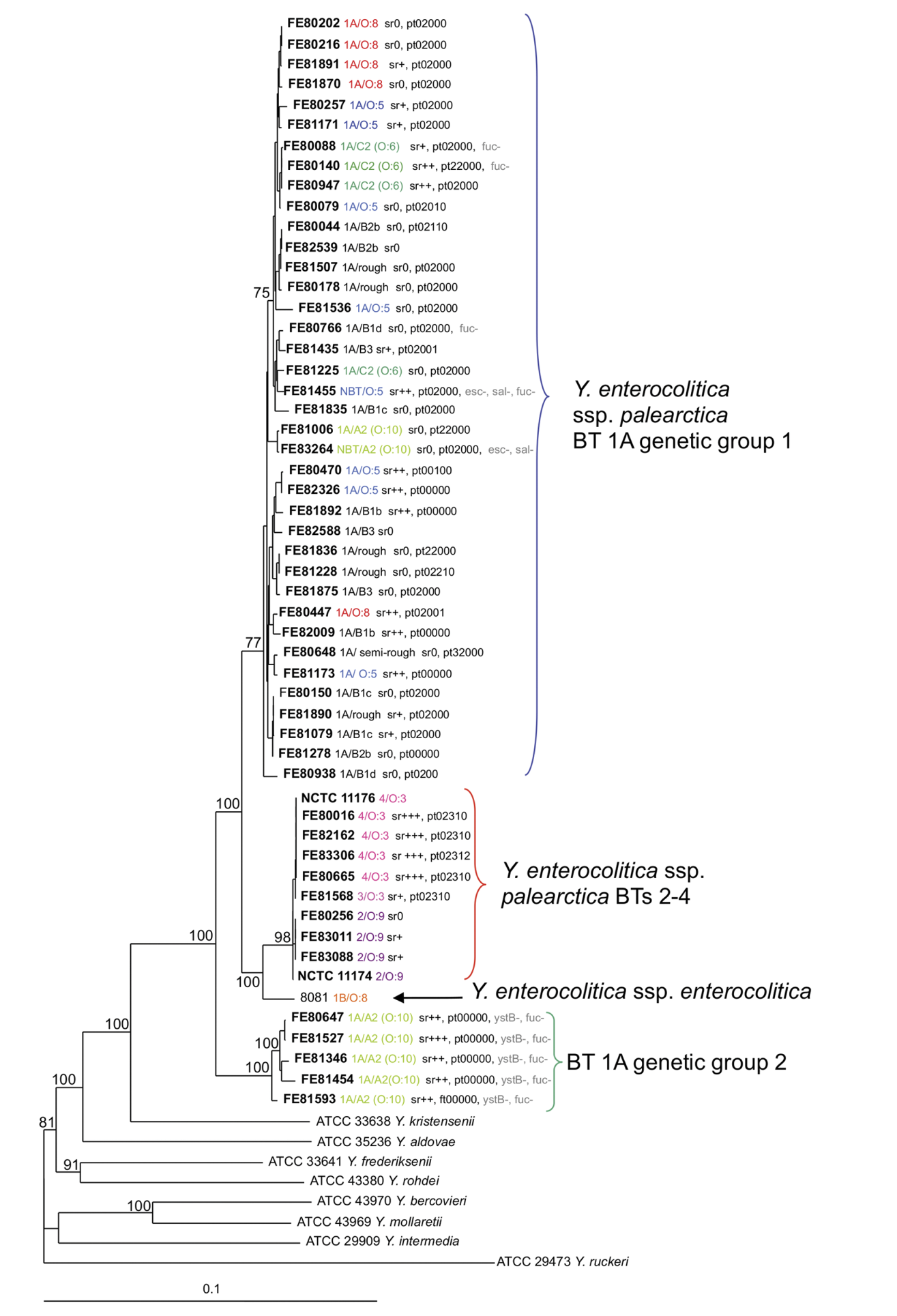


**Additional figure 1. Neighbour-joining tree based on seven concatenated MLST genes (4580 bp).** Neighbour-joining bootstrap confidence values over 75% (1000 replicates) are given in the branches. BT 1A strains were *ystB* positive in PCR and had positive reaction in fucose fermentation unless otherwise indicated. sr=serum resistance; pt= phage type, which encodes reaction to 5 phages (φR1–37, PY100, φYeO3–1, φR1-RT, φ80–81). Strains sequenced in the present study are marked bold. In addition, the following GenBank sequences were used: *Y. enterocolitica* 8081 (AM286415), *Y. aldovae* ATCC 35236 (ACCB00000000), *Y. kristensenii* ATCC 33638: (ACCA00000000), *Y. intermedia* ATCC 29909 (AALF00000000), *Y. frederiksenii* ATCC 33641 (AALE00000000), *Y. mollaretii* ATCC 43969 (AALD00000000), *Y. bercovieri* ATCC 43970 (AALC00000000), *Y. rohdei* ATCC 43380 (ACCD00000000) and *Y. ruckeri* ATCC 29473 (ACCC00000000).
