# Supplementary material for: Clinical isolates of Yersinia enterocolitica Biotype 1A represent two phylogenetic lineages with differing pathogenicity-related properties
Source: BMC Microbiol. 2012 Sep 17;12:208. doi: 10.1186/1471-2180-12-208 (PMC3512526; doi:10.1186/1471-2180-12-208)
Supplement: Additional 2 — Analysis of Y. enterocolitica LPS by DOC-PAGE and silver staining. The picture is compiled of gel images with different LPS types as indicated above the lanes by the LPS type codes that are explained in the text box. Please note that LPS types A2, B1c, B1d, B2a, B2c and B4 are not shown. The gel regions where O-PS and lipid A (LA) plus core migrate are indicated by arrows. [file 1471-2180-12-208-S2.docx]

**Additional file 2. Lipopolysaccharide typing of the *Yersinia* isolates.**

Type A LPS had a homopolymeric O-PS that is demonstrated by the smear in the high-molecular weight region of the gel (Figure A1). Type B LPS had a ladder-forming and type C a single-length heteropolymeric O-PS while type D LPS lacked an O-PS or had a lipid A core substituted with a single O-repeat unit (Figure A1). The strains with type A LPS were separated into subtypes A1, A2 and A3 based on migration of the homopolymer smear in the gel. The ladder-forming type B strains separated into subtypes B1–B4, with most of the analysed strains falling into subtype B2. Subtypes B1 and B2 both had two main steps in the ladder, the difference being that the distance between the steps was longer in subtype B2. In addition, among some B1 and B2 strains the presence of a weak third ladder step or a homopolymeric smear was observed (Figure A1). These minor differences were used to classify the strains to subgroups B1a-B1d and B2a-B2d. Subtype B3 ladders had typically 5–6 main steps where some modality could be detected. Subtype B4 ladders had typically more than 10 steps and a very distinct modality pattern. Subtypes C1 and C2 were observed for LPS type C; C1 had a shorter O-PS with an estimated 15 sugar residues, while that of C2 was longer with estimated 30 residues. One third of all the analysed strains belonged to subtype C1, and 45 strains to C2. Twelve strains were missing an O-PS or had a lipid-A core substituted with a single O-repeat unit and therefore were classified as type D (rough or semi-rough).


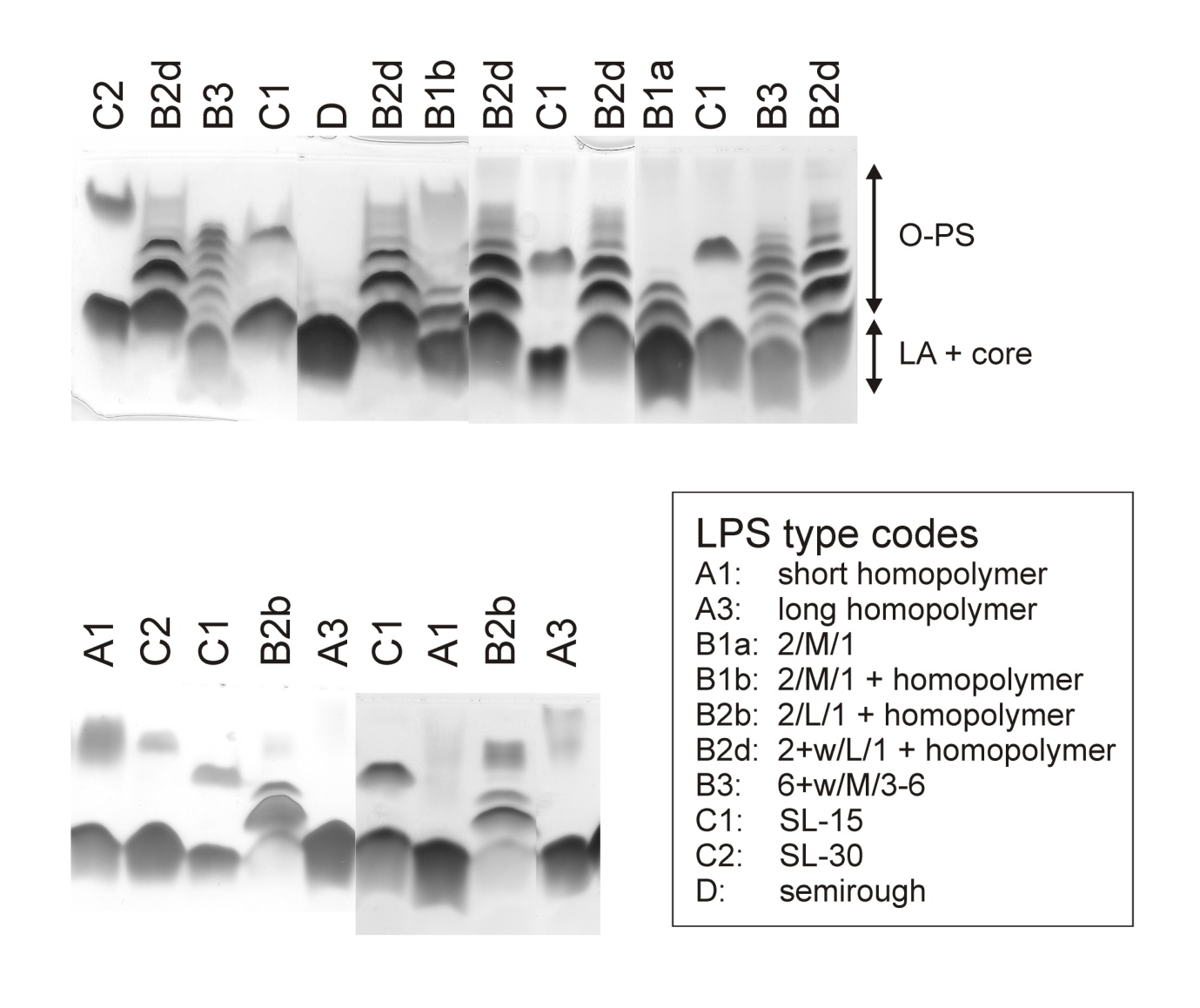


**Additional figure 2. Analysis of *Y. enterocolitica* LPS by DOC-PAGE and silver staining.** The picture is compiled of gel images with different LPS types as indicated above the lanes by the LPS type codes that are explained in the text box. Please note that LPS types A2, B1c, B1d, B2a, B2c and B4 are not shown. The gel regions where O-PS and lipid A (LA) plus core migrate are indicated by arrows.
